# The role of the dorsolateral prefrontal cortex in control of skin sympathetic nerve activity in humans

**DOI:** 10.1093/cercor/bhad112

**Published:** 2023-05-04

**Authors:** Rebecca Wong, Gianni Sesa-Ashton, Sudipta Datta, Brendan McCarthy, Luke A Henderson, Tye Dawood, Vaughan G Macefield

**Affiliations:** Baker Heart and Diabetes Institute, Melbourne, VIC, Australia; Baker Department of Cardiometabolic Health, The University of Melbourne, VIC, Australia; Baker Heart and Diabetes Institute, Melbourne, VIC, Australia; Baker Heart and Diabetes Institute, Melbourne, VIC, Australia; Baker Department of Cardiometabolic Health, The University of Melbourne, VIC, Australia; Baker Heart and Diabetes Institute, Melbourne, VIC, Australia; Baker Department of Cardiometabolic Health, The University of Melbourne, VIC, Australia; School of Medical Sciences (Neuroscience), Brain and Mind Centre, The University of Sydney, NSW, Australia; Baker Heart and Diabetes Institute, Melbourne, VIC, Australia; Baker Department of Cardiometabolic Health, The University of Melbourne, VIC, Australia; Baker Heart and Diabetes Institute, Melbourne, VIC, Australia; Baker Department of Cardiometabolic Health, The University of Melbourne, VIC, Australia; Department of Anatomy and Physiology, The University of Melbourne, VIC, Australia; Department of Neuroscience, Central Clinical School, Monash University, VIC, Australia

**Keywords:** Dorsolateral prefrontal cortex, Medullary raphe, Transcranial electrical stimulation, Sympathetic nervous system

## Abstract

The dorsolateral prefrontal cortex (dlPFC) is primarily involved in higher order executive functions, with there being evidence of lateralization. Brain imaging studies have revealed its link to the generation of skin sympathetic nerve activity (SSNA), which is elevated in states of emotional arousal or anxiety. However, no studies have directly explored dlPFC influences on SSNA. Transcranial alternating current stimulation (−2 to 2 mA, 0.08 Hz, 100 cycles) was applied between the left or right dlPFC and nasion via surface electrodes. Spontaneous bursts of SSNA were recorded from the common peroneal nerve via a tungsten microelectrode in 21 healthy participants. The modulation index was calculated for each stimulation paradigm by constructing cross-correlation histograms between SSNA and the sinusoidal stimulus. Stimulation of the dlPFC caused significant modulation of SSNA, but there was no significant difference in the median modulation index across sides. Stimulation also caused cyclic modulation of skin blood flow and sweat release. We have shown for the first time that stimulation of the dlPFC causes modulation of SSNA, also reflected in the effector-organ responses. This supports a role for the dlPFC in the control of SSNA, which likely contributes to the ability of emotions to bring about cutaneous vasoconstriction and sweat release.

## Introduction

The dorsolateral prefrontal cortex (dlPFC) is widely known for its role in executive functions, which encompass planning, cognitive flexibility, and working memory (Grafman et al. 2005; Rougier et al. 2005; Diamond 2013; Jones and Graff-Radford 2021). Recently, our laboratory demonstrated that transcranial alternating current stimulation (tACS) of the dlPFC in conscious human participants led to partial entrainment of muscle sympathetic nerve activity (MSNA), blood pressure, and heart rate (Sesa-Ashton et al. 2022). These findings imply that the dlPFC may serve an important role in cardiovascular control. The dlPFC is also involved in the top-down control of emotional expression (Amidfar et al. 2019; Sanchez-Lopez et al. 2021). Many markers of emotional expression are brought about by changes in skin sympathetic nerve activity (SSNA), which controls the release of sweat, the diameter of cutaneous blood vessels, erects the hairs, and liberates fatty acids from subcutaneous adipose tissue (Macefield and Wallin 1996; Macefield and Wallin 1999; Glatte et al. 2019).

Direct recordings of SSNA can be measured via microneurography (Macefield 2021). This is an invasive but safe procedure that involves the percutaneous insertion of a microelectrode into a peripheral nerve of awake humans. When coupled with functional magnetic resonance imaging (fMRI), this enables the identification of brain regions associated with the generation of SSNA (Macefield and Henderson 2016). Past imaging studies have suggested that the dlPFC and SSNA may be linked. Henderson et al. (2012) observed that spontaneous fluctuations in SSNA were correlated with the fluctuations in Blood Oxygen Level Dependent (BOLD) signal intensity in the precuneus and the frontal cortex. Indeed, the precuneus is the most metabolically active area of the brain and is considered the cornerstone of consciousness (Cavanna and Trimble, 2006); it also has extensive links to the frontal cortex, which includes the dlPFC (Zhang and Li 2012). In addition, James et al. (2013) observed that there was a positive correlation between SSNA and dlPFC activity when participants were subjected to emotionally charged pictures. This correlation may be due to the dlPFC’s direct link to the amygdala, the primary role of which is to process emotions (Swanson and Petrovich, 1998). Thus, it can be assumed that an association between the dlPFC and SSNA exists, and that the dlPFC may be able to modulate SSNA.

Given that we had shown that tACS of the dlPFC modulates MSNA (Sesa-Ashton et al. 2022), it is not unreasonable to suggest that the same approach may also reveal modulation of SSNA. Transcranial alternating current stimulation involves delivering a biphasic, sinusoidal current to a specific region of the scalp via two surface electrodes, in which the anode during the first half of the stimulus cycle becomes the cathode during the second half (Woods et al. 2016). The cyclic stimulation produces transient and slight changes in brain function by causing physiological entrainment of cortical activity, thus influencing brain functions (Colzato et al. 2017; Tavakoli and Yun 2017). To this end, we used the same procedure to determine whether sinusoidal stimulation of the ipsilateral dlPFC modulates SSNA and effector organs in the skin. We also assessed whether lateralization exists by stimulating the contralateral dlPFC in a subset of participants.

## Methods

This study comprised 21 healthy participants (11 females and 10 males) ranging in age from 21 to 38 years. A complete set of data was obtained from 12 participants, which included the data from stimulating the right and left side of the dlPFC. For the remaining 9 participants, data were only obtained during stimulation of the right dlPFC. This study was approved by the Human Research Ethics Committee of Western Sydney University (research protocol H11010), endorsed by Governance at The Baker Heart and Diabetes Institute, and conformed to the Declaration of Helsinki. All participants provided informed written consent and refrained from caffeinated food and beverages, as well as nicotine, for at least 4 h before the experiment.

### Recording SSNA

Participants were seated with their legs in the extended position on a slightly reclined chair. A vacuum casting cushion (Germa Protec, Sweden) was placed underneath the thigh of the right leg for stabilization. Recording of SSNA began by searching for the common peroneal nerve via palpation of the leg at the fibular head. The segment of the nerve closest to the skin’s surface was mapped via external electrical stimulation (0.2 ms, 1–10 mA, 1 Hz). The optimal site for stimulating the nerve, which generated the strongest sensations of paraesthesia, was sterilized with an alcohol swab. A 200 μm diameter, insulated tungsten microelectrode (Frederick Haer and Co, Bowdoin, ME, United States) was manually inserted through the skin. An uninsulated reference microelectrode was also inserted ~1 cm away at a depth of 1–2 mm. The two microelectrodes were connected to an isolated amplifier and headstage (NeuroAmp EX, ADInstruments, Sydney, Australia), which amplified and filtered the neural activity detected (gain 2 × 10^4^, bandpass 0.3–5.0 kHz). Neural activity was stored on a computer using a software-controlled data acquisition and analysis system (PowerLab 16SP hardware and LabChart for Macintosh, v7.2.5 software; ADInstruments). The stimulating microelectrode was manually directed into a cutaneous fascicle by systematically adjusting its angle and depth while delivering weak electrical pulses (0.2 ms, 0.01–1.0 mA, 1 Hz). When a current of ≤ 0.02 mA evoked paraesthesia, the microelectrode tip was considered to have penetrated a cutaneous fascicle, and the stimulating leads were disconnected. The identity of a cutaneous fascicle was confirmed by the absence of nerve impulses during the passive stretching of muscles innervated by the common peroneal nerve. It was also confirmed by the generation of afferent nerve impulses by stroking the skin of the fascicular innervation territory on the dorsum of the foot or the side of the leg. Spontaneous bursts of SSNA were identified by their long bursts and responses to brisk sniffs or an unexpected arousal stimulus. The presence of SSNA was confirmed by observing the RMS-processed signal that was displayed in real-time, which showed sympathetic bursts as distinct peaks. Additionally, infrared photoelectric plethysmography probes (MLT1020, ADInstruments, Sydney, Australia), which detected changes in pulsatile skin blood volume, were attached to the pad of the left big toe. Ag–AgCl electrodes were placed on the pads of digits II and III of the left foot to measure spontaneous or evoked sweat release (GSR Amplifier, AD Instruments, Australia).

### tACS stimulation protocols

An electroencephalogram (EEG) cap was placed over the head and EEG positions F3 and F4, indicating the left and right dlPFC, respectively, were marked. After removing the cap, Ag–AgCl surface electrodes were placed over these two sites; a similar electrode was placed on the nasion. To stimulate the left dlPFC, the F3 and nasion electrodes were used as the anode and cathode (the orders of which were reversed during each cycle of stimulation). Similarly, to stimulate the right dlPFC, the F4 and nasion electrodes were used. A total of 100 cycles of the sinusoidal stimuli, ranging from −2 to 2 mA at a frequency of 0.08 Hz were generated by the data-acquisition system (PowerLab 16SP hardware) and delivered using an isolated current stimulator (Linear Stimulus Isolator, World Precision Instruments, Sarasota, United States). Physiological measurements were obtained in response to one set of stimuli, either the right (ipsilateral to the nerve recording) or left (contralateral to the nerve recording) dlPFC, in random order. An interval of 3 to 5 min was placed before the other stimulation protocol began, with each set of stimuli lasting ~ 21 min.

### Data analysis

Analysis was conducted primarily on the negative-going spikes of the raw nerve recordings. Negative-going spikes and the positive peaks of the sinusoidal stimulation were detected via discriminator software on LabChart 7 Pro (Spike Histogram for Macintosh v2.5.1, ADInstruments, Sydney, Australia). These were used to create autocorrelation and cross-correlation histograms. For every participant, an autocorrelation histogram of the positive sine wave peaks applied onto the right dlPFC and nasion, and a cross-correlogram of the same sine wave peaks and SSNA were made. A second pair of histograms was also created from subjects who had their left dlPFC stimulated. This involved making an autocorrelation histogram (50 ms bins) of the positive sine wave peaks applied onto the left dlPFC and nasion, and a cross-correlation histogram (50 ms bins) of the same sine wave peaks and SSNA. To analyze the histograms created, they were exported as a text file and pasted into a statistical analysis program (GraphPad Prism 7 for Windows v7.03, GraphPad Software, United States). Here, the text file was converted into bar graphs, and second-order polynomials were utilized to fit smooth curves to the cross-correlation histograms. From the smoothed curve, the cyclic modulation of SSNA during stimulation of the dlPFC was quantified by measuring the modulation index. This was determined by finding the number of spikes between the peak and trough at a time that is closest to zero, and calculating the modulation index from the following formula: Modulation index (%) = [(peak—trough)/peak] × 100.

Since two peaks of SSNA for each sinusoidal cycle of stimulation were observed in most cross-correlation histograms, they were also assessed and their latencies were evaluated. The larger of the two peaks was dubbed the primary peak, while the smaller one was called the secondary peak. The primary and secondary peak latencies were calculated using identical methods; that is, the time elapsed from time zero to the peak.

Mean sweat release and skin perfusion were sampled between the sixth R-wave before each peak of the sinusoidal stimulation to the sixth R-wave after each stimulation. Each participant’s data were normalized using the sixth R-wave before the peak as a baseline at 100%. Bar graphs were constructed from this, and the peaks and troughs of each graph were used to determine if stimulation of the dlPFC affects blood perfusion and sweat release. It was calculated by following this formula: Percentage change (%) = [(peak—trough)/peak] × 100.

**Fig. 1:**
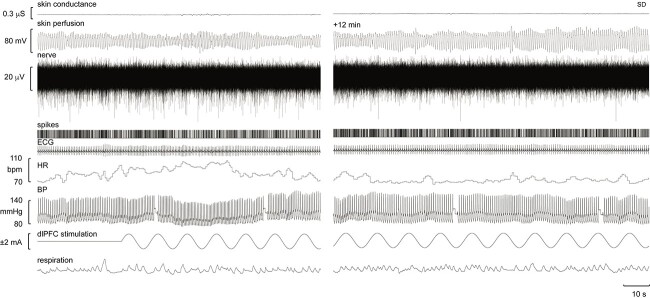
Multi-unit recording of SSNA, together with electrical skin conductance, skin perfusion, electrocardiographic activity (ECG), heart rate (HR), blood pressure (BP) and respiration at rest and during tACS over the ipsilateral dlPFC. Discriminated, negative-going (sympathetic) spikes extracted from the nerve recording are illustrated as standard pulses (spikes). The selection in the right panel, obtained 12 min into the stimulation, shows that bursts of SSNA continued during the stimulation.

Prism 9.0 for Macintosh (GraphPad Software) was utilized for statistical analyses. D’Agostino and Pearson Normality tests were performed on the dataset to test for normality. To examine whether modulation of SSNA by the dlPFC occurred, the modulation indices calculated from each cross-correlogram depicting dlPFC stimulation and SSNA were compared with no modulation using the Mann–Whitney test. The same test and significance levels were also utilized to determine whether there was a difference between: (1) the primary and secondary peak latencies during ipsilateral stimulation; (2) the primary and secondary peak latencies during contralateral stimulation; (3) ipsilateral and contralateral stimulation on primary peak latency; and (4) ipsilateral and contralateral stimulation on secondary peak latency. To examine whether sweat and blood flow can be influenced by the dlPFC, a one-sample t-test was utilized on each participant’s mean sweat release and blood perfusion data, and compared with the theoretical value of 0, representing no change. The significance level chosen was *P* < 0.05 and data are presented as median and percentiles.

## Results

Successful recordings were obtained from the right common peroneal nerve in 19 participants; for two participants recordings were obtained from the left nerve. Stimulation of the dlPFC did not cause any sensations other than slight tingling under the scalp electrodes that abated over a few minutes. Experimental records from one participant during stimulation of the ipsilateral dlPFC are shown in Fig. 1. It can be seen that this participant had high levels of SSNA in anticipation of the start of the stimulation, which caused a further increase that subsided as the stimulation continued for ~21 min. While there were clear fluctuations in skin blood flow, as measured by the pulsatile changes in skin perfusion, this participant showed a negligible increase in sweat release during stimulation. The negative-going sympathetic spikes have been extracted from the nerve signal and represented as standard pulses. Together with event markers for the peak of the sinusoidal stimulus, these timing events were used to construct cross-correlation histograms between the SSNA and the sinusoidal stimulus applied to the dlPFC.

### Modulation of SSNA during dlPFC stimulation

**Fig. 2:**
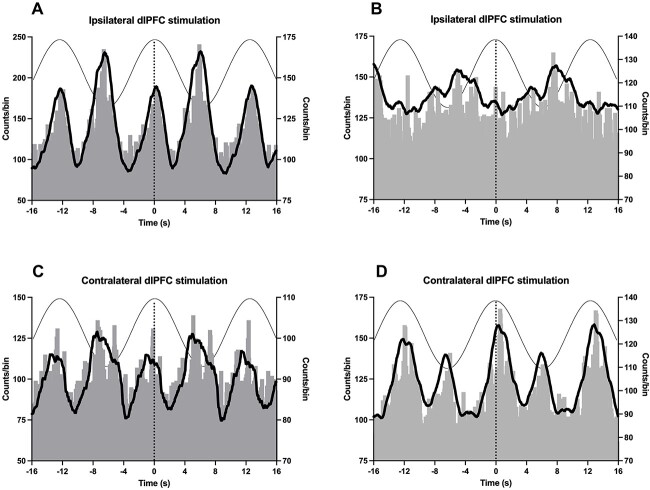
Cross-correlation histograms showing the modulation of SSNA by sinusoidal stimulation of the ipsilateral (**A**, **B**) and contralateral (**C**, **D**) dlPFC in two participants (**A**, **C** and **B**, **D**). A smoothed polynomial (thick, black line) was fitted to the data, illustrating peaks of SSNA modulation. The left vertical axis refers to counts per 50 ms bin of the cross-correlogram, while the right axis refers to the mean counts of the smoothed polynomial, hence the different scales. Stimulation of the dlPFC is depicted by the sinusoidal wave at the top of the graph.

Cross-correlation analysis revealed clear cyclic modulation of SSNA during sinusoidal stimulation of the ipsilateral dlPFC, as shown in the examples from two participants in Fig. 2. Most cross-correlation histograms exhibited two distinct peaks. They occurred at different time points during a single sinusoidal cycle and were referred to as the primary and secondary peaks for the larger and smaller peaks, respectively (Fig. 2). The primary peaks of the cross-correlation histograms occurred either close to the peak or the trough of the stimulating sine wave. Their location appeared to differ according to the side of dlPFC stimulation: ipsilateral stimulation appeared to generate primary peaks that were more associated with the trough of the sinusoidal stimulus than contralateral stimulation, but this was not statistically significant (76% vs. 58%, respectively; *P* = 0.45). Measured from the positive peak of the sinusoidal stimulus, there was no significant difference between ipsilateral and contralateral stimulation of the dlPFC on latency of the primary (*P* = 0.21) or secondary (*P* = 0.52) peak.

Calculated from the primary peak, the median modulation index observed for ipsilateral stimulation of the dlPFC was 9.2% (*n* = 21); the corresponding value for stimulation of the contralateral side was 14.0% (*n* = 12). When all data were combined, the modulation of SSNA was statistically significant when compared with a modulation index of 0 (*P* < 0.01). Individual modulation indices, calculated from the primary peaks, for all participants are presented in Table 1. While there was a greater range of modulation indices with ipsilateral stimulation, there was no significant difference in the magnitude of modulation between the two sides of stimulation (*P* = 0.34); this did not change when the two participants in whom SSNA was recorded from the opposite (left) leg were removed from the dataset (*P* = 0.41).

**Table 1:** Median and 95% confidence intervals for modulation index of primary peak and latency of primary peak, measured from the positive peak of the sinusoidal stimulus.

	**Ipsilateral dlPFC stimulation**	**Contralateral dlPFC stimulation**
**Modulation index of primary peak**	Median = 9.17 [7.50, 26.32] Range = 89.00 *n* = 21	Median = 13.98 [9.50, 21.86] Range = 27.39 *n* = 12
**Latency of primary peak**	Median = 6.25 [5.50, 8.18] Range = 10.80 *n* = 21	Median = 4.625 [1.00, 7.08] Range = 12.00 *n* = 12

Although there were no differences in the magnitudes and latencies of the primary and secondary peaks with ipsilateral or contralateral stimulation, there were differences in the complexity of the cross-correlograms. This was indicated by the morphology of the primary and secondary peaks according to the side of stimulation. Most of those produced during contralateral stimulation of the dlPFC were clearer, with a well-defined separation of peaks compared with the majority of those produced during ipsilateral stimulation (83% vs. 43%, *P* = 0.03; Mann–Whitney test); examples are shown in Fig. 2.

### Modulation of skin blood flow and sweat release during dlPFC stimulation

**Fig 3:**
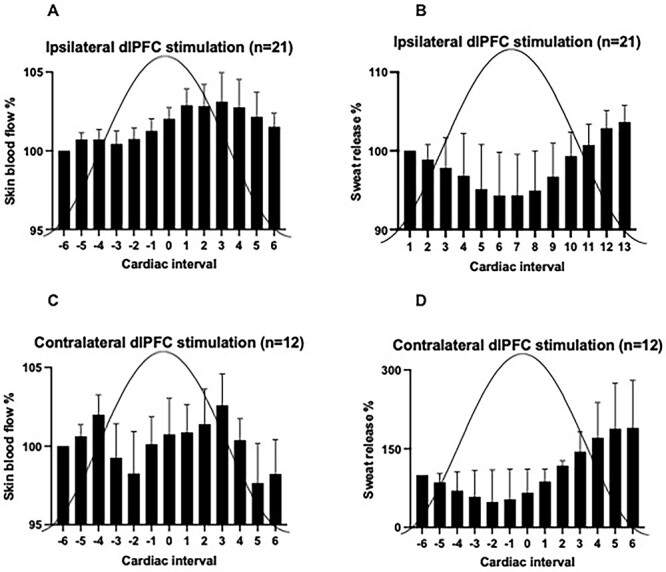
Cyclic changes in skin blood flow (**a**, **c**) and sweat release (**B**, **D**) in response to dlPFC stimulation. Mean data are shown over six cardiac intervals before and six cardiac intervals after the peak of the sinusoidal stimulus applied to the ipsilateral (**A**, **B**) and contralateral (**C**, **D**) dlPFC. Error bars show the standard error of the mean.

Cyclic fluctuations in skin blood flow and sweat release were apparent during both ipsilateral and contralateral dlPFC stimulation, as shown in Fig. 3. Compared with zero modulation, the modulation of skin blood flow was statistically significant for both ipsilateral (*P* = 0.0001) and contralateral (*P* = 0.0017) stimulation of the dlPFC, whereas ipsilateral (*P* = 0.0103) but not contralateral (*P* = 0.1510) stimulation caused a significant modulation of sweat release.

## Discussion

This study has shown, for the first time, that sinusoidal electrical stimulation of the dlPFC causes a significant cyclic modulation of SSNA, skin blood flow, and sweat release in awake humans. We had recently shown that dlPFC stimulation causes a cyclic modulation of MSNA, together with a cyclic modulation of heart rate and blood pressure, but no change in respiration (Sesa-Ashton et al. 2022). Both of these studies emphasize the role of higher cortical areas in the control of sympathetic outflow to muscle or skin, and move our understanding of the operation of the dlPFC beyond its classical roles in executive functions.

We had previously shown that sinusoidal galvanic vestibular stimulation (sGVS), a means of selectively altering the firing of vestibular afferents, causes cyclic modulation of SSNA together with illusions of side-to-side motion and, in some individuals, nausea (James et al. 2010; El Sayed et al. 2012; Hammam et al. 2012). Participants who reported motion sickness had greater modulation of SSNA, but not of MSNA (Hammam et al. 2012; Klingberg et al. 2015). Using the same frequency and intensity of stimulation (0.08 Hz, −2 to 2 mA) as employed in these previous studies, we have now shown that sinusoidal stimulation of the dlPFC also modulates SSNA, with a median modulation index of 12%. This is about one-third the magnitude of the modulation produced by sGVS (32%), and—unlike sGVS—stimulation of the dlPFC does not induce any illusions of motion.

Two peaks of modulation—the larger primary peak and the secondary smaller peak—were typically observed within a single cycle of sinusoidal stimulation. It is hypothesized that the generation of these peaks was related to the cyclic fluctuation in membrane potentials of neurones within the dlPFC (Hammam et al. 2011; Hammam et al. 2012). Except for two experiments, SSNA was always recorded from the right leg, and most primary peaks occurred when the right (ipsilateral) dlPFC was depolarized during the negative (cathodal) phase of the sinusoidal stimulation. At the same time, the nasion, which acted as the anode, was hyperpolarized during the positive phase of the stimulus. As for the secondary peak, most were generated when the ipsilateral dlPFC was hyperpolarized during the positive peak. Surprisingly, contralateral stimulation of the dlPFC had a greater likelihood of generating more distinct peaks of modulation, in which the cross-correlation histograms were less ambiguous than those generated by ipsilateral stimulation. Presumably, this reflects the cross-over of motor (and sensory) pathways between the brain and spinal cord, although little is known about the decussation of sympathetic outflow (Harrison et al. 1939).

Lateralization has been shown to occur when performing complex executive tasks, allowing efficiency to be improved through specialization of the left and right dlPFC (Kaller et al. 2011). For example, when one is planning a set of tasks, the left side of the dlPFC oversees the details of separate events, while the right side of the dlPFC integrates these events into a coherent sequence (Grafman et al. 2005; Kaller et al. 2011). However, there was no significant difference in the magnitude of modulation of SSNA between stimulation of the left and right dlPFC. Lateralization may not have been evident because the dlPFC was not performing complex executive tasks during the experiment; participants were simply instructed to relax during dlPFC stimulation.

There is increasing evidence indicating that tACS does affect brain function (Colzato et al. 2017; Tavakoli and Yun, 2017; Elyamany et al. 2021). Moreover, delivering tACS while performing fMRI of the brain has revealed that tACS induces changes in BOLD signal intensity that are not limited to areas immediately under the stimulating electrodes; rather some of the strongest changes occur in remote areas, emphasizing the idea that tACS acts to modulate networks within the brain rather than acting exclusively on local areas (Cabral-Calderin et al. 2016). It is likely that electrical stimulation of the dlPFC acts via projections to specific nuclei in the hypothalamus, and from there to the midbrain and, ultimately, to the medullary raphe. The medullary raphe is believed to be the final output nucleus for the generation of cutaneous vasoconstrictor drive through two mechanisms: a serotonergic pathway that activates 5-HT2A receptors located on sympathetic preganglionic neurones, and a glutamatergic pathway (Ootsuka and Tanaka 2015). Individuals with mood disorders, including major depressive disorder, anxiety disorder, obsessive–compulsive disorder and panic disorder, have been shown to have disruptions in their serotonergic pathways (Maron and Shlik 2005; Van Dijk et al. 2008; Lin et al. 2014; Jenkins et al. 2016).

*Limitations*

Most studies that use transcranial electrical stimulation (tDCS, tACS) of the dlPFC apply direct (tDCS) or alternating (tACS) current between electrode sites F3 and F4. In our study we applied sinusoidal current between an electrode over the left (F3) or right (F4) dlPFC and the nasion, such that the current would likely also have influenced the ventromedial prefrontal cortex (vmPFC). Moreover, due to interconnectivity between the dlPFC and vmPFC (Hare et al. 2014; Saraiva and Marshall 2015; Schmidt et al. 2018), modulation of SSNA during stimulation of the dlPFC may have occurred—in whole or in part—via the vmPFC. Strong evidence of the dlPFC and vmPFC’s close connectivity during the performance of tasks has been provided by many studies, including decision-making based on value and context, and self-control (Hare et al. 2014; Saraiva and Marshall 2015; Schmidt et al. 2018). Our previous neuroimaging studies have shown that, like the dlPFC, the vmPFC is correlated with SSNA when observing emotionally charged images (Henderson et al. 2012), suggesting that the vmPFC may also be involved in the modulation of SSNA. Finally, it is also worth pointing out that electrical stimulation of the scalp causes skin sensations (tingling, burning) under the electrodes, and that the sensations caused by delivering transcranial direct current stimulation (tDCS) for 20 minutes increases the excitability of trigemino-facial reflexes (Cabib et al. 2016). However, in our experience, any cutaneous sensations are transient: we always ask participants what they felt after the stimulation and a common report is that the tingling/burning sensations disappeared after a couple of minutes. Thus, we do not believe that continuous stimulation of cutaneous afferents under the scalp electrodes are responsible for the effects observed during tACS.

## Conclusions

Overall, the dlPFC was shown to modulate SSNA which highlights, for the first time, a novel function of the dlPFC independent of its role in executive functions. Given that elevated SSNA features in states of elevated arousal and anxiety, leading to increases in sweat release and cutaneous vasoconstriction (Brown et al. 2012), modulation of the dlPFC may lead to clinical applications. This includes the development of treatment options for those with disorders characterized by elevated SSNA, such as hyperhidrosis (Macefield et al. 2008). It also emphasizes how disruption of the serotonin pathway in mood disorders may lead to elevated levels of SSNA (Maron and Shlik 2005; Van Dijk et al. 2008; Lin et al., 2014; Jenkins et al. 2016; Francescangeli et al. 2019).

## CRediT authors statement

Rebecca Wong (Formal analysis, Investigation, Visualization, Writing—original draft, Writing—review & editing), Jonathon Sesa-Ashton (Investigation, Writing—review & editing), Sudipta Datta (Investigation, Writing—review & editing), Brendan McCarthy (Investigation, Writing—review & editing), Luke Henderson (Conceptualization, Writing—review & editing), Tye Dawood (Conceptualization, Investigation, Methodology, Project administration, Supervision, Writing—review & editing), Vaughan G Macefield (Conceptualization, Data curation, Formal analysis, Investigation, Methodology, Resources, Software, Supervision, Visualization, Writing—review & editing).

## Funding

This work was supported by a Senior Principal Research Fellowship awarded to Vaughan Macefield by the Baker Heart and Diabetes Institute.

*Conflict of interest statement*: All authors declare no conflict of interests.Data availability statement: The data underlying this article will be shared on reasonable request to the corresponding author.

## Data availability statement

The data underlying this article will be shared on reasonable request to the corresponding author.
